# Castration causes an increase in lysosomal size and upregulation of cathepsin D expression in principal cells along with increased secretion of procathepsin D and prosaposin oligomers in adult rat epididymis

**DOI:** 10.1371/journal.pone.0250454

**Published:** 2021-04-29

**Authors:** Lorena Carvelli, Andrea Carolina Aguilera, Leila Zyla, Laura Lucía Pereyra, Carlos R. Morales, Louis Hermo, Miguel A. Sosa

**Affiliations:** 1 Laboratorio de Biología y Fisiología Celular “Dr. Francisco Bertini”, Instituto de Histología y Embriología—CONICET—Facultad de Ciencias Médicas—Universidad Nacional de Cuyo, Mendoza, Argentina; 2 Facultad de Ciencias Exactas y Naturales, Universidad Nacional de Cuyo, Mendoza, Argentina; 3 Department of Anatomy and Cell Biology, McGill University, Montreal, Québec, Canada; University of Hyderabad, INDIA

## Abstract

In the epididymis, lysosomal proteins of the epithelial cells are normally targeted from the Golgi apparatus to lysosomes for degradation, although their secretion into the epididymal lumen has been documented and associated with sperm maturation. In this study, cathepsin D (CatD) and prosaposin (PSAP) were examined in adult epididymis of control, and 2-day castrated rats without (Ct) and with testosterone replacement (Ct+T) to evaluate their expression and regulation within epididymal epithelial cells. By light microscope-immunocytochemistry, a quantitative increase in size of lysosomes in principal cells of Ct animals was noted from the distal initial segment to the proximal cauda. Androgen replacement did not restore the size of lysosomes to control levels. Western blot analysis revealed a significant increase in CatD expression in the epididymis of Ct animals, which suggested an upregulation of its expression in principal cells; androgens restored levels of CatD to that of controls. In contrast, PSAP expression in Ct animals was not altered from controls. Additionally, an increase in procathepsin D levels was noted from samples of the epididymal fluid of Ct compared to control animals, accompanied by an increased complex formation with PSAP. Moreover, an increased oligomerization of prosaposin was observed in the epididymal lumen of Ct rats, with changes reverted to controls in Ct+T animals. Taken together these data suggest castration causes an increased uptake of substrates that are acted upon by CatD in lysosomes of principal cells and in the lumen by procathepsin D. These substrates may be derived from apoptotic cells noted in the lumen of proximal regions and possibly by degenerating sperm in distal regions of the epididymis of Ct animals. Exploring the mechanisms by which lysosomal enzymes are synthesized and secreted by the epididymis may help resolve some of the issues originating from epididymal dysfunctions with relevance to sperm maturation.

## Introduction

For more than half a century, the mammalian epididymis has attracted the attention of reproductive biologists due to its role in sperm maturation [1, 2]. While structurally formed in the testis, spermatozoa acquire the ability to become motile and fertile as they transit through the epididymal duct [3]. Here, they encounter a luminal environment that is unique for each of its major regions, i.e. initial segment, caput, corpus and cauda, as determined in part by the secretory and endocytic functions of its epithelial cells [1, 4]. While several distinct cell types form the epithelium, principal cells are the most abundant, narrow/apical, clear and basal cells constitute lower numbers [1, 4, 5]. By balancing the secretion and endocytosis of proteins, ions and other substances, an epididymal luminal milieu is created that mediates the proper sequence of biochemical and molecular modifications for transforming sperm into a fertile and motile state [6, 7].

In addition to secretion, the endocytosis of proteins from the lumen occurs as a major function of epithelial clear cells, with principal cells also demonstrating endocytosis but to a lesser degree [1, 4, 6, 8]. The requirements for receptor mediated endocytosis involve organelles of the endocytic apparatus consisting of clathrin-coated pits, early and late endosomes, and eventually lysosomes [1, 9–11]. Lysosomal enzymes derived from the Golgi apparatus are targeted to lysosomes by specific receptors located on small approximately 100nm vesicles [12] and where endocytosed proteins are degraded to fine tune the epididymal luminal environment [11].

One of the lysosomal proteins that inhabits lysosomes of epididymal epithelial cells is the aspartic endopeptidase cathepsin D (CatD) [13–15], with numerous physiological functions demonstrated for this protein, including activation and degradation of polypeptide hormones, growth factors and regulation of programed cell death [16, 17]. In some cell types, CatD is transported from the *trans*-Golgi Network (TGN) to lysosomes as the 52kDa precursor procathepsin D (proCatD) via specific mannose-6-phosphate receptors (MPR) [18] and this also appears to occur in epididymal principal cells [19]. Upon entering the acidic lysosomal compartment, proCatD (52 kDa) is processed and yields an approximate 44–48 kDa intermediate that eventually gives rise to a dimer of disulfide-linked heavy (~31–34) and light (14 kDa) chains [16, 20]. In some physiological and pathological conditions, proCatD escapes the normal targeting mechanism and is secreted from cells into the extracellular environment of different tissues [21, 22], with its presence also detected in the epididymal lumen [19, 23].

Another protein present in lysosomes of epithelial cells of the epididymis is the sphingolipid hydrolase activator precursor, prosaposin (PSAP), previously named sulfated glycoprotein-1 (SGP-1) [14, 24, 25]. PSAP is broken down in lysosomes into 4 sphingolipd activator proteins, saposins A, B, C and D required for the hydrolysis of glycosphingolipids [26]. PSAP is commonly targeted selectively to lysosomes through sortilin receptor [27], but it can also be secreted into the extracellular spaces of various tissues [26, 28] including the lumen of the testis and epididymis [24]. Interestingly, oligomerization of PSAP (linked covalently by intermolecular disulphide bridges) is crucial for its entry into the secretory pathway [29].

Modifications of glycoproteins and lipids on the sperm plasma membrane is an essential consideration for sperm maturation [1, 30, 31]. In addition to the secretion of proteins added to the sperm surface, a loss or modification of glycoproteins occurs due to the presence of acid glycosidases, glycosyl transferases and other enzymes in the epididymal lumen [2, 32, 33]. Currently, one of the accepted concepts is that the epididymis favours the catalytic activity of luminal enzymes [34, 35], producing significant biochemical changes in sperm [33–36].

An intriguing aspect of epididymal functions is its dependence on the testis for proper maintenance of epithelial cell integrity and sperm maturation. Castration, resulting in absence of all testicular factors including hormonal androgens arriving via the circulation causes dramatic changes to epididymal epithelial cells [8, 37, 38], including their apoptosis [39–41]. In addition to major secretory proteins being affected, studies have reported that synthesis and intracellular targeting of acid hydrolases to lysosomes is altered in castrated rats [35, 42]. Castration also induces an increase in secretion of proCatD in the rat cauda epididymidis [15].

Ligation of the efferent ducts (EDL) selectively blocks substances emanating from the testis and entering the epididymal lumen via the efferent ducts. The absence of these substances consisting of Sertoli cell derived proteins, sperm, high intraluminal androgens, estrogens, collectively termed lumicrine factors, leads to profound effects on the epididymal morphology and functions, especially in the initial segment [43–46].

Based on numerous studies on the epididymis as an androgen-dependent organ [1, 46–49], we examined the effects of castration on CatD and PSAP expression in lysosomes of principal cells, as well as the secretion of proCatD and PSAP into the epididymal lumen. This study was done on normal versus 2-day castrated adult rats supplemented with or without testosterone in combination with light microscope-immunohistochemistry (LM-IHC), western blot and co-immunoprecipitation analyses.

Exploring the mechanisms by which lysosomal proteins respond to insults such as castration may help resolve some of the issues originating from epididymal dysfunctions with relevance to sperm maturation.

## Materials and methods

### Reagents

The polyclonal goat anti-cathepsin D (cat.sc-6486) antibody was purchased from Santa Cruz Biotechnology. The polyclonal rabbit anti-prosaposin was produced and characterized by Dr. C. R. Morales (McGill University, Montreal, Canada) [50]. The HRP-conjugated anti-goat IgG antiserum was obtained from Merck Millipore, (cat.401515, Calbiochem ® Burlington, MA, USA) and the HRP-conjugated anti-rabbit IgG was purchased from Sigma (A9169, St. Louis, MO). Chemiluminescent reagents (ECL, Pierce) were obtained from Thermo Scientific (32106, Inc. Rockford, IL, USA). LM-IHC of proteins was performed using the Envision + peroxidase diaminobenzidine kit (K4010, DakoCytoimation kit, Mississauga, Canada).

### Controls, castration and testosterone replacement

Adult Sprague-Dawley male rats (90 day old and weighing 300-350g, provided by the IHEM-CONICET animal facility, Mendoza, Argentina), maintained under standard conditions (food and water *ad libitum* at 20–22°C and 12h:12 h light:dark cycle), were divided into three groups (n = 3 per group): controls, castrated and castrated with testosterone replacement. Experimental procedures were carried out following the method of other authors with slight modifications [35]. Briefly, all animals were anesthetized with an abdominal injection of ketamine hydrochloride (70 mg/kg) and xylazine (5 mg/kg). Thereafter, the testes and epididymides of 3 rats were exposed, and both the testicular vessels and efferent ducts were ligated without compromising the blood supply to the latter and epididymides. After removal of the testes, the epididymides were placed back into the scrotum. In addition, three other castrated rats at the time of surgery were injected intraperitoneally with testosterone (1 mg/kg each injection in corn oil as vehicle), and another three control rats with the vehicle alone according to the protocol of Fan and Robaire [40]. After 48h, the animals of all experimental groups were sacrificed, and the epididymides processed as detailed below.

All experimental procedures were reviewed and approved by the animal care and use committee of School of Medicine, Universidad Nacional de Cuyo (Institutional Committee for the Care and Use of Laboratory Animals, CICUAL; protocol reference number: 175/2019, http://fcm.uncuyo.edu.ar/paginas/index/cicual), in strict accordance with the recommendations in the Guide for the Care and Use of Laboratory Animals: Eighth Edition. Washington, DC: The National Academies Press. https://doi.org/10.17226/12910.

### Quantification of lysosomal size in principal cells

Epididymides processed by LM-IHC for CatD expression were used to calculate the lysosomal profile area of principal cells from control, castrated and castrated with testosterone replacement rats (n = 3 in each case). Cross section images of tubules of the epididymis from the distal initial segment and proximal caput designated as the IS/caput region and the distal corpus and proximal cauda designated as the corpus/cauda region regions were taken with a Nikon Eclipse 80i (provided with a Nikon DS-Fi1-U3 digital camera) at 60x magnification (1280x960 pixels) and analysed using ImageJ software (1.53a version, National Institute of Health, USA. http://imagej.nih.gov/ij). The corresponding setting of the scale was carried out with an equivalent of 132 pixels per 20um. The profile area of 30 principal cells from the IS/caput and corpus/cauda from each experimental treatment and control animals (10 per animal, n = 3) was delineated as well as their lysosomes in the cytoplasm. The profile area of the cell and its lysosomes (μm2) were measured and subjected to statistical analysis.

### Processing of biological material for immunoblotting

Epididymides of control (n = 3 rats) and experimental animals (castrated and castrated with testosterone replacement, n = 3 rats each group) were divided into three regions; initial segment/caput, corpus and cauda, with each region being processed separately. Samples from both epididymides of each animal were pooled. In each case the epididymis was cut into small pieces with stainless steel blades and suspended (1:3 w/v) in Hank’s buffer at 32°C for 30 min with gentle manual agitation. The minced epididymides were sedimented for 10 min at 4°C, and the resulting supernatant (S1; containing epididymal fluid and sperm) extracted and kept on ice. Tissues were washed twice with Hank’s buffer followed by mixing with S1 and centrifugation at 1000 x g for 10 min. The pellets containing spermatozoa were separated from the supernatants (epididymal fluid). The remaining epididymal tissue was weighed and suspended in (1:5 w/v) buffer H (10 mM Tris-acetate, pH 7.2, containing 0.25 M sucrose, 0.25 M EDTA, 1 mM PMSF, 0.02% sodium azide and 5 mM glycerophosphate) and homogenized. The homogenates were centrifuged at 800 x g for 20 min at 4°C, and the resulting postnuclear supernatants were stored at -20°C until use, along with epididymal fluids.

### Electrophoresis (SDS-PAGE) and immunoblotting

Proteins from epididymal fluid or tissue (45 μg) from control (n = 3 rats) and experimental animals (castrated and castrated with testosterone replacement, n = 3 rats each group) obtained as described above were solubilized and analyzed in SDS-PAGE gels (8–10% acrylamide) according to the method of Laemmli [51] and electrotransferred to 0.45 μm pore-nitrocellulose membranes (GE10600002, GE Healthcare, Amersham, Germany), according to Burnette [52]. The processing of membranes for the detection of proteins was similar to Alberdi et al. [53]. Briefly, after blocking with 5% milk, the membranes were incubated for 12 h at 4°C with either anti-cathepsin D (1:1000) or anti-prosaposin (1:5000) antibodies, all diluted in PBS-T (10 mM NaH_2_PO_4_-Na_2_HPO_4,_ pH 7.2, PBS, containing 0.05% (v/v) Tween 20). The membranes were then washed three times with PBS-T and incubated with corresponding HRP-conjugated secondary antibodies (1:5000) for 2 hours. Finally, after three washes with PBS-T, the specific bands were detected by a chemiluminescence method (Pierce Biotechnology Inc.) and the signal was detected with a LAS 4000 imaging system (Fujifilm Lifescience, USA). Band intensities were quantified by densitometric scanning, using an Image J software (Image Processing and Analysis in Java; National Institutes of Health, Bethesda, MD, USA). For detection of PSAP oligomers, epididymal fluid proteins from either control, castrated or castrated with testosterone replacement were solubilized in Laemmli sample buffer without the reducing agent (dithiotreitol), and run in SDS-PAGE gels (7% acrylamide). After transferring on nitrocellulose membranes PSAP was detected with anti-prosaposin as described above.

Ponceau S (P3504, St. Louis, MO) staining was used as a loading control in all cases. Stain was performed for 20 seconds with 0.1% Ponceau S (w/v) in 5% acetic acid solution.

### Light microscopic-immunohistochemistry

Epididymides of each control (n = 3 rats) and experimental animals (castrated and castrated with hormone replacement, n = 3 rats for each group) were removed and immediately fixed in Bouin’s solution for 72 hours, dehydrated and embedded in paraffin according to the method of Hermo et al. [54]. Thick sections (5 μm) were cut and mounted on glass slides. After blocking with Peroxidase Block (Dako Cytoimation) the sections were incubated for 12 h with either anti-cathepsin D (1:200) or anti-prosaposin (1:300) antibody diluted in buffer from DakoCytoimation (S0809, Mississauga, Canada). The anti-goat peroxidase-conjugated antibodies (1:250 dilution) and the anti-rabbit (K4010, DakoCytoimation kit) were used as secondary antibodies. Immunolocalization of proteins was performed using the Envision+ peroxidase diaminobenzidine (DAB) kit (K4010, DakoCytoimation kit) [54]. Preimmune rabbit or goat serum was used as control of antibody specificity. All digital images from the major regions of epididymis (initial segment, caput, corpus and cauda) were taken with a Nikon Eclipse 80i (digital camera: Nikon DS-Fi1-U3).

### Immunoprecipitation

Epididymides from control (n = 3 rats) and experimental animals (castrated and castrated with hormone replacement, n = 3 rats for each group) were processed similarly to those for immunoblotting as described above, with the epididymal fluid being used for this assay. Commercial sepharose beads conjugated with protein A (Amersham Bioscience) were washed twice with 1 ml PBS (phosphate buffered saline) and centrifuged at 3000 x g for 3 min. The beads were resuspended in 100 μl of PBS and incubated with 2 μg of anti-prosaposin antibody for 2 h at 4°C. After incubation, the beads were centrifuged at 3000 x g for 5 min and washed twice with PBS. The beads were resuspended in 100 μl of PBS and incubated for 16 h at 4°C with 1 ml of epididymal fluid after which they were spun down at 3000 x g for 5 min and washed twice with PBS. The proteins bound to the beads were solubilized with Laemmli buffer, analyzed by SDS-PAGE and electrotransferred to nitrocellulose membranes (GE10600002, GE Healthcare, Amersham, Germany). The co-immunoprecipitated anti-cathepsin D was detected by immunoblotting as detailed above. Detection of rabbit IgG (heavy chain) in the precipitates was used as loading control.

### Statistical analysis

Statistical calculations were performed using GraphPad Prism software (GraphPad software Inc., USA). P-values were calculated using Tukey’s Multiple Comparisons Test. The level of significance was set at p≤0.05 and p≤0.01. At least three independent experiments were performed in each case.

## Results

### Expression and distribution of cathepsin D in epithelial cells of the caput, corpus and cauda regions in control and 2-day castrated animals

**Fig 1:**
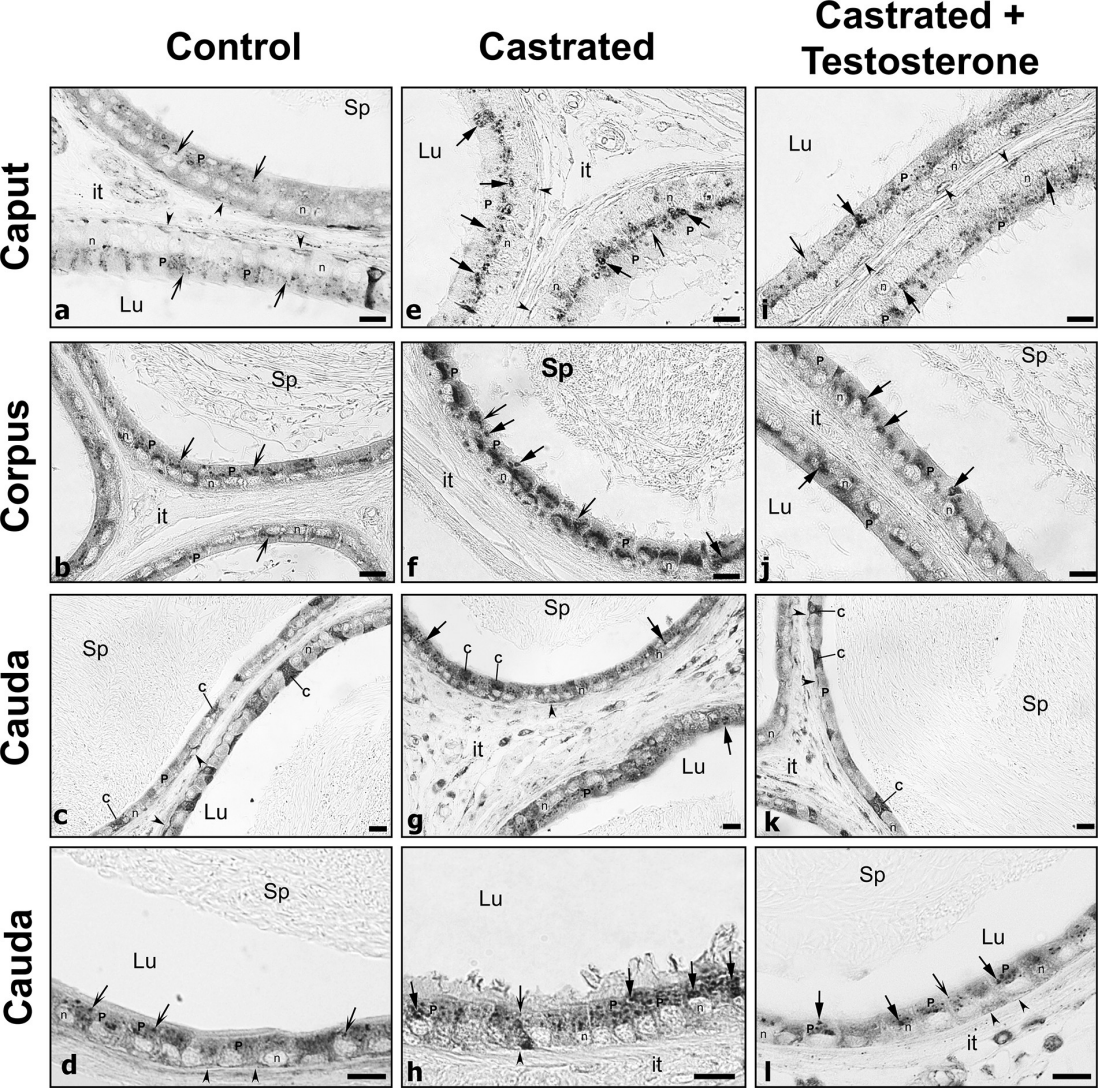
Immunostaining of epididymal tubules with cathepsin D of caput, corpus and cauda regions from control (Con), castrated (Ct) and castrated with testosterone supplementation (Ct+T) animals. Small dense spherical reactive lysosomes (arrows with winged head) of principal cells (P) are evident in (Con) animals. In (Ct) animals, lysosomes (arrows with triangular head) appear to be larger in size and more abundant, with some remaining large in (Ct+T) animals. Some of these lysosomes show a dense homogeneous appearance, while others show a densely stained ring enveloped by a pale central core (triangular arrows). Lu, lumen; it, interstitial space; Sp, spermatozoa; reactive basal (arrowheads) and clear cells (C); n, nuclei of principal cells. Scale bars = 20 μm.

**Fig 2:**
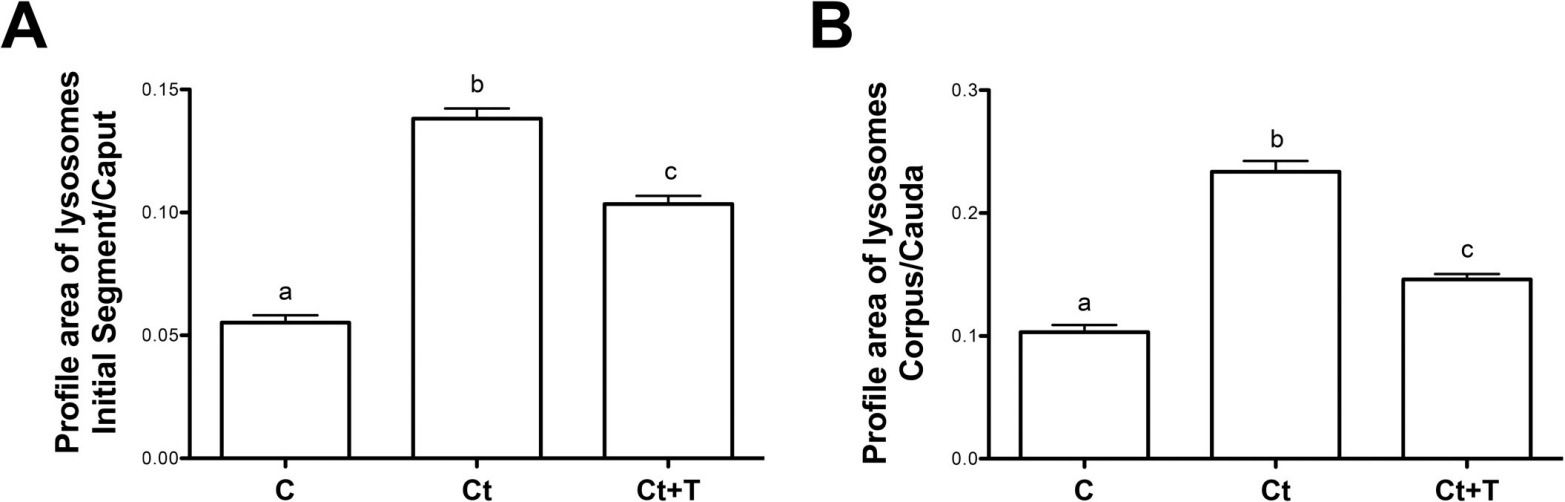
Quantification of lysosomal profile areas of principal cells. Lysosomal profile areas were estimated from digital images obtained by immunocytochemical studies for control (Con), castrated (Ct) and castrated with testosterone supplementation (Ct+T) animals. Calculations were performed on principal cells of the distal IS and proximal caput designated as the IS/Caput region and the distal corpus and proximal cauda designated as the corpus/cauda region. Bars represent the means of lysosomal profile area per cell ± SEM from three independent experiments for IS/caput (A) and corpus/cauda (B) regions. a, b and c are significantly different from each other (p < 0.0001, obtained by Tukey’s Multiple Comparisons Test).

In control animals, CatD by LM-IHC was distributed supranuclearly in principal cells of the caput, corpus and cauda regions, as spherical reactive bodies with a homogeneous dense staining appearance and of relatively small size and abundance (Fig 1A–1D). Such structures were deduced to be lysosomes as documented by electron microscope (EM) cytochemical studies of our lab [13, 14]. After castration, CatD reactive lysosomes appeared to be increased in size (Fig 1E–1H). Furthermore, while the size of lysosomes was enhanced, some revealed a dense peripheral ring of reaction product enveloping a central pale stained core (Fig 1E–1H). Quantitatively, the difference in size was confirmed in castrated animals with significant statistical values in both of the 2 regions examined, i.e. IS/caput and corpus/cauda, as compared to control values (Fig 2A and 2B).

Replacement of testosterone to castrated animals did not rescue lysosomal size to control levels (Fig 2A and 2B), nor was the appearance of lysosomes identical to controls (Fig 1I–1L).

Clear cells, the dominant endocytic epithelial cell under normal conditions, are larger and broader cells than the adjacent principal cells [1, 4, 8] and are especially numerous in the cauda region (Fig 1C, 1G and 1H). CatD expression appeared as a dense reactive product throughout the entire cytoplasm of clear cells, as noted with different antibodies [11, 55] and which is consistent with the plethora of lysosomes observed in these cells by EM analysis [1, 4]. However, no qualitative difference in the intensity of reactivity or shape and size of clear cells was noted between control and experimental animals.

Basal cells lodged at the base of the epithelium and presenting thin processes that radiate along the basement membrane were reactive but with no apparent changes observed in their reactivity or appearance between control and experimental animals (Fig 1A–1L).

### Expression and distribution of cathepsin D in the initial segment

The initial segment (IS) has been defined as a region characterized by specific regulating factors emanating from the testis, referred to as lumicrine factors [57–60].

**Fig 3:**
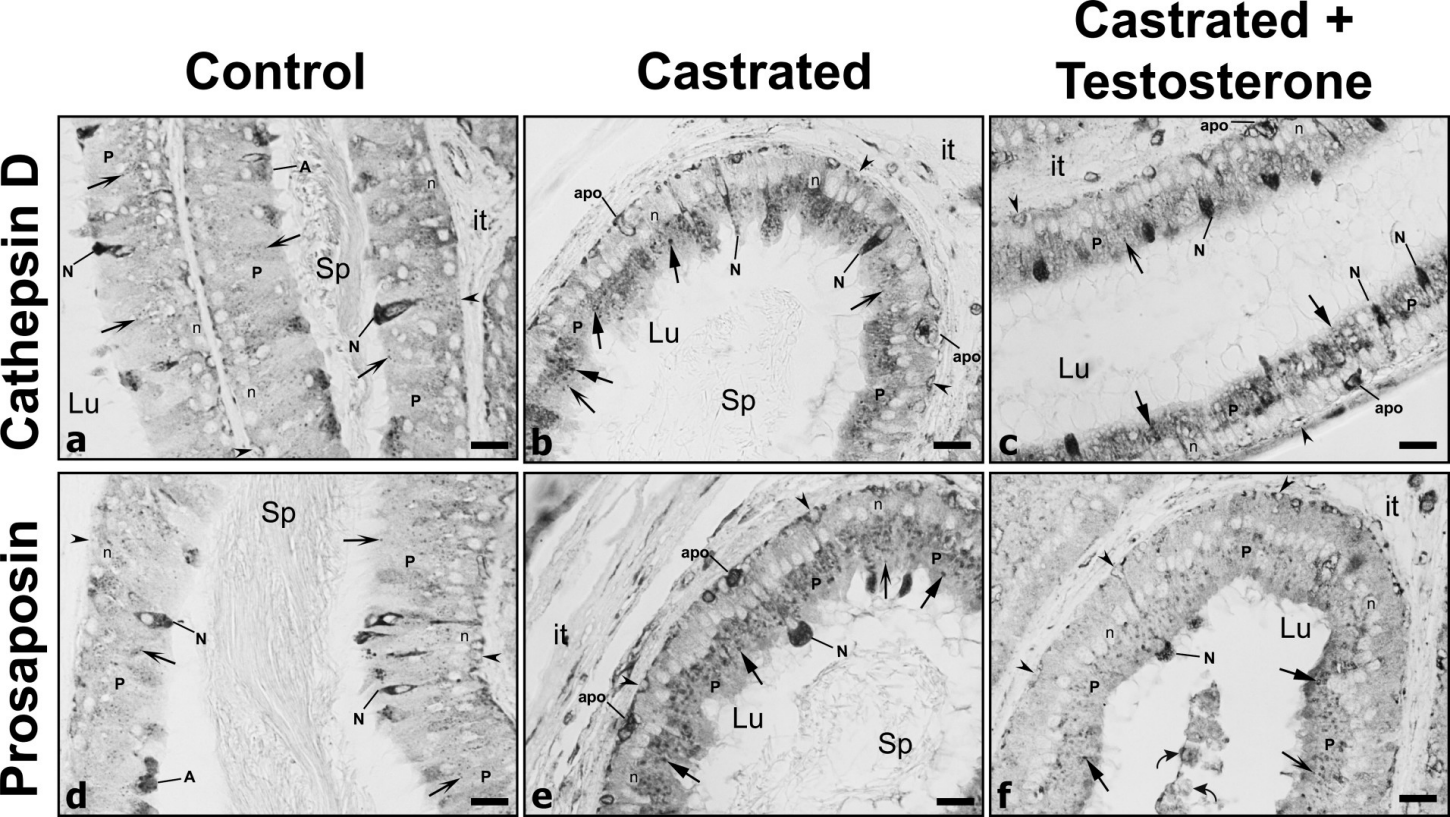
Immunostaining of epididymal tubules with cathepsin D and prosaposin of the initial segment from control (Con), castrated (Ct) and castrated with testosterone supplementation (Ct+T) animals. Small dense reactive spherical lysosomes (winged arrows) are seen in principal cells (P) of (Con) animals. However, lysosomes appear to be more abundant and larger in size in (Ct) and (Ct+T) animals with both antibodies, with some revealing a pale central core surrounded by a ring of dense reactivity (triangular arrows). Few basally located apoptotic bodies (apo) are evident in epithelium of (Ct) and (Ct+T) animals. The lumen of a (Ct+T) animal contains cellular debris (curved arrows). Lu, lumen; it, interstitial space; Sp, spermatozoa; reactive basal (arrowheads), narrow (N) and apical (A) cells; n, nuclei of principal cells. Scale bars = 25 μm.

With respect to CatD expression, reactive lysosomes in principal cells of this region in control animals were of small size and spherical with a dense content (Fig 3A). After castration, lysosomes were large (Fig 3B) with some revealing a dense ring of reaction product enveloping a central pale stained core. Such lysosomes were also evident in castrated animals supplemented with testosterone (Fig 3B). Quantitatively, a statistically significant difference was noted in the size of principal cell lysosomes in the IS/caput region both in the absence (Fig 2A) and presence (Fig 2B) of testosterone as compared to controls. Thus the situation for lysosomal size is similar in the initial segment as for the other epididymal regions.

Narrow/apical cells that occupy this region of the duct [1, 4, 5] were highly reactive for CatD but did not display any differences in their reactivity under each experimental condition (Fig 3A–3C). Basal cells were also reactive for CatD in control and experimental animals (Fig 3A–3C), with a few appearing to be undergoing apoptosis (Fig 3B and 3C). Importantly, after castration CatD expression in narrow/apical and basal cells was maintained, even in the absence of all testicular factors.

### Western blot analysis of cathepsin D expression in whole epididymal tissue and epididymal luminal fluid

**Fig 4:**
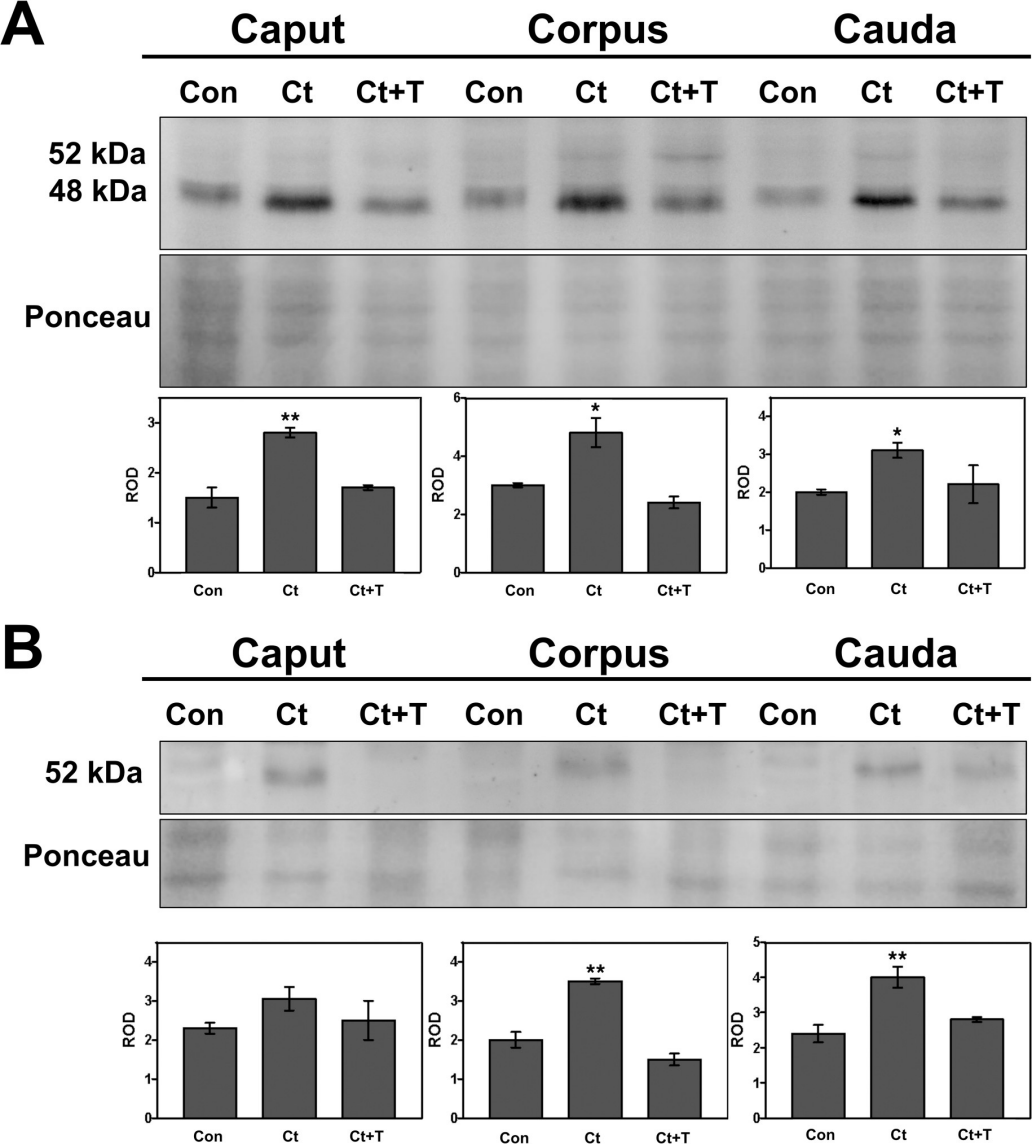
Immunodetection of cathepsin D in whole epididymal tissue (A) and luminal fluid (B) of the epididymis from control (Con), castrated (Ct) and castrated with testosterone supplementation (Ct+T) animals. Three regions of the epididymis were examined, i.e. IS/caput, corpus and cauda. Bands of 52 kDa and 48 kDa corresponding to immature and intermediate forms of CatD, respectively, are indicated. The figure shows a representative immunoblot for each region and experimental condition and the quantification of the bands in each case (intermediate CatD for tissues and immature form in fluids). Bars represent the means of relative optical density (ROD) ± SE from three independent experiments. (*) and (**) are significant differences from the control (p < 0.05 and p < 0.01 respectively, obtained by Tukey’s multiple comparisons Test). Ponceau staining was used as loading control.

The data from western blot immunoblotting of whole adult epididymal tissue (Fig 4A) revealed a significant increase of CatD levels observed as an intermediate form of 48 kDa in castrated animals of the three regions (Fig 4A, upper panel) correlating with the increase in size of lysosomes of principal cells caused by an enhancement of the endocytic process of these cells (Fig 2A and 2B). Administration of testosterone to castrated animals reversed the situation of CatD expression to control levels in all epididymal regions (Fig 4A, lower panel) suggesting that testosterone fine-tunes the levels of CatD expression in lysosomes of principal cells in control animals but not their size.

Western blot immunoblotting of the epididymal luminal fluid revealed an increase of the CatD precursor, the 52 kDa proCatD (Fig 4B, upper panel), with statistical significance differences noted for the corpus and cauda regions (Fig 4B, lower panel). The effects of castration were reversed by testosterone replacement, which returned levels to that of controls (Fig 4B, lower panel).

### Expression and distribution of prosaposin

**Fig 5:**
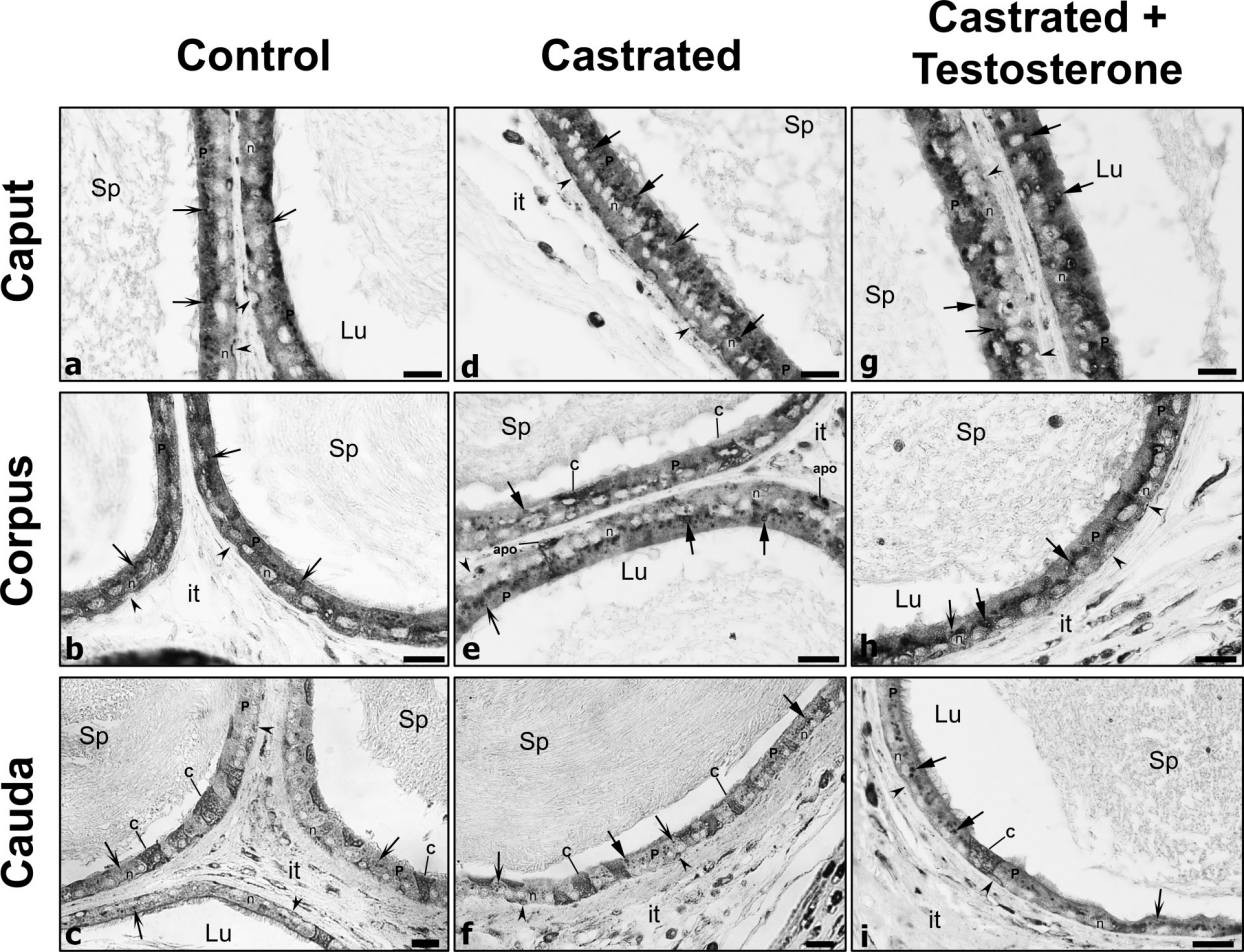
Low power of epididymal tubules of caput, corpus and cauda regions from control (Con), castrated (Ct) and castrated with testosterone supplementation (Ct+T) animals immunostained with prosaposin. While small reactive spherical lysosomes (winged arrows) appear in principal cells (P) of all 3 groups, in (Ct) animals, lysosomes (triangular head) appear to be larger in size and more abundant, with some remaining large in (Ct+T) animals. Some of these lysosomes show a dense homogeneous appearance, while others show a densely stained ring enveloped by a pale central core (triangular arrows). Apo, apoptotic basal cell; Lu, lumen; it, interstitial space; Sp, spermatozoa; reactive basal (arrowheads) and clear (C) cells; n, nuclei of principal cells. Scale bars = 20 μm.

**Fig 6:**
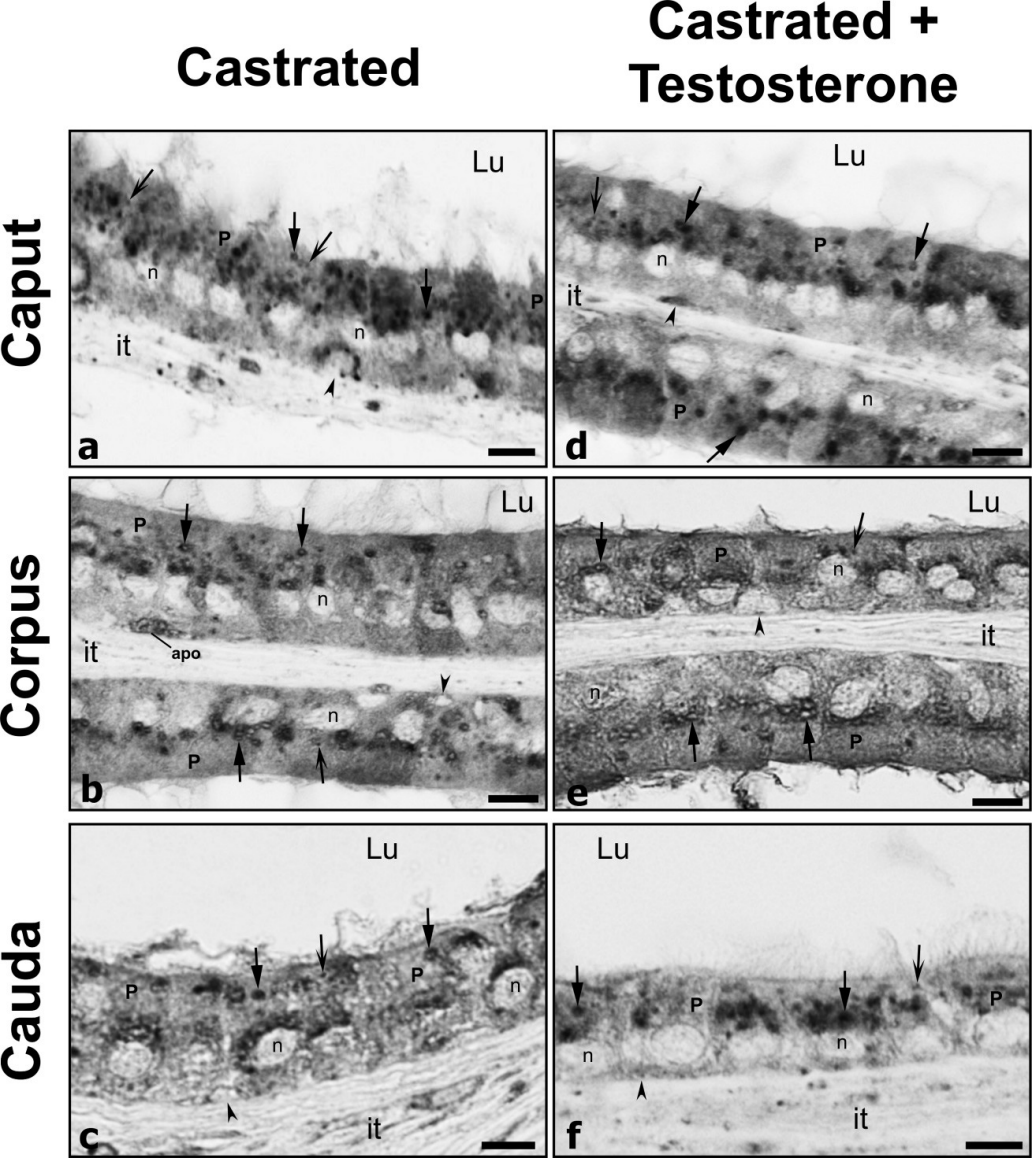
High power of epididymal tubules of caput, corpus and cauda regions from castrated (Ct) and castrated with testosterone supplementation (Ct+T) animals immunostained with prosaposin. In both (Ct) and (Ct+T) animals, lysosomes (arrows with triangular head) within principal cells (P) appear to be larger in size and more abundant. Some of these lysosomes show a dense homogeneous appearance, while others show a densely stained ring enveloped by a pale central core (triangular arrows). Small reactive spherical lysosomes (winged arrows) also appear in principal cells (P) of all 3 groups. Apo, apoptotic basal cell; Lu, lumen; it, interstitial space; reactive basal cells (arrowheads); n, nuclei of principal cells. Scale bars = 10 μm.

Given that prosaposin (PSAP) has been implicated in the targeting and activation of CatD [56, 57], the status of the latter was assessed in the present study. By LM-IHC, PSAP expression in control animals was noted as small punctuate reactive spherical lysosomes located supranuclearly in principal cells of the initial segment (Fig 3D), caput, corpus and cauda regions (Fig 5A–5D). Such reactive bodies were deduced to be lysosomes as demonstrated by our previous EM cytochemical studies [13, 14]. Qualitatively, lysosomes in principal cells of these regions appeared to be increased in size in castrated animals (Figs 3E, 3F, 5E–5H and 6A–6C) compared to controls, as verified by quantification of CatD reactive lysosomes (Fig 2A and 2B). Some of these large lysosomes revealed a dense ring of reactivity enveloped by a central pale stained core (Figs 3E, 5E, 5F, 5H and 6A–6C). Administration of testosterone did not alleviate the presence of these large lysosomes (Figs 3F, 5I, 5J, 5L and 6D–6F) suggesting that factors other than testosterone maintain the normal status of the size and appearance of principal cells, as noted above for expression of CatD under similar conditions.

Narrow/apical cells were also reactive for PSAP in controls and experimental animals (Fig 3D–3F), as were clear cells with reactivity extending throughout the entire cytoplasm and being comparable to that noted in castrated animals with or without testosterone replacement (Fig 5C, 5G and 5K). Basal cells were also reactive under different experimental conditions (Figs 3D–3F and 5A–5L), with a few appearing to undergo apoptosis after castration (Fig 3E). Importantly, after castration, PSAP expression in principal cells and these other epithelial cells was maintained, even in the absence of all testicular factors.

The observations under these different experimental conditions for both CatD and PSAP were consistent on numerous epididymal tissue sections as obtained from 3 different animals for each of the experimental groups. Control experiments employing normal rabbit and goat serum revealed a complete absence of reaction, similar to our previous studies on CatD and PSAP expression in the epididymis [13, 14, 25].

### Western blot analysis of PSAP expression in whole epididymal tissue and epididymal luminal fluid

**Fig 7:**
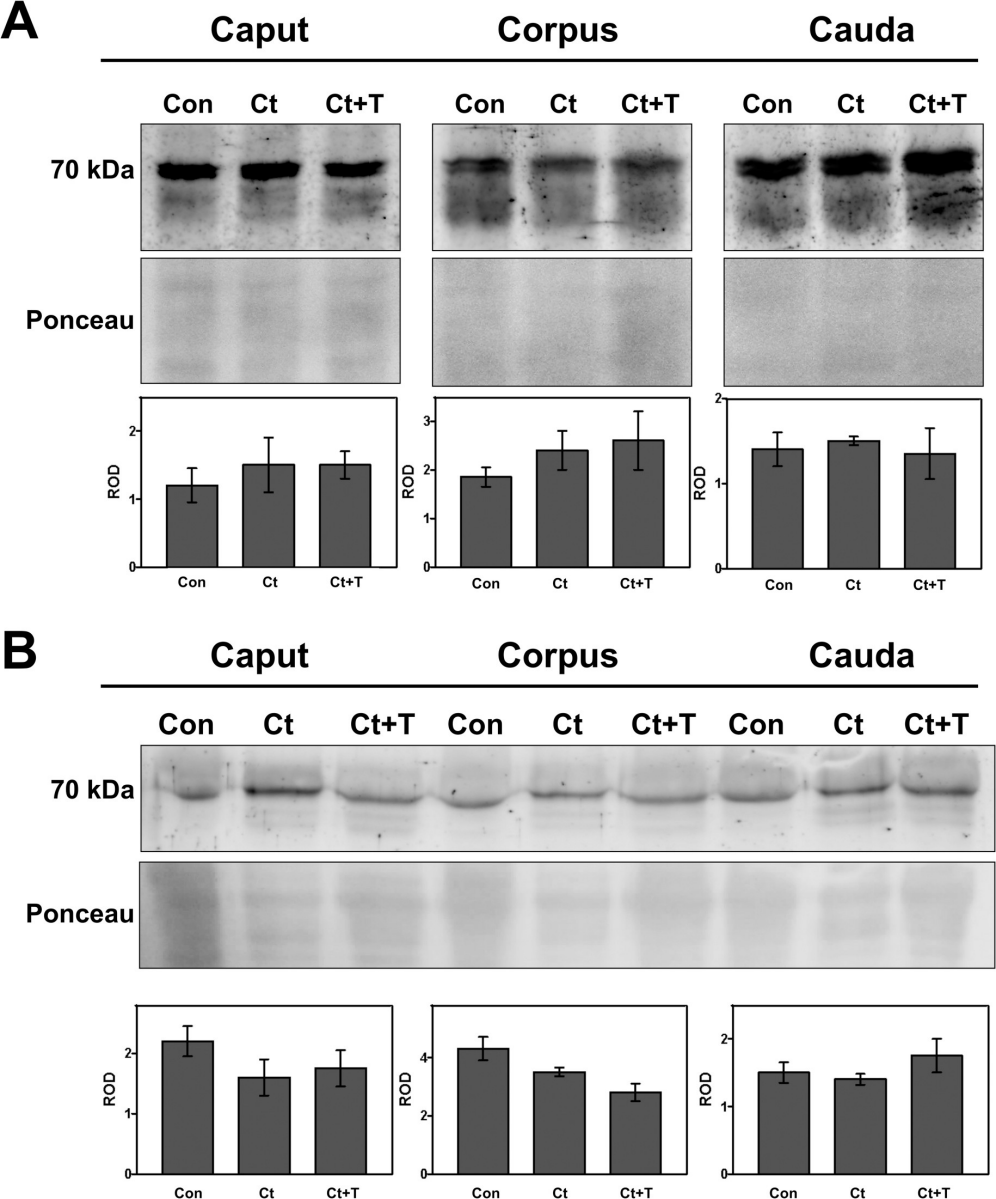
Immunodetection of prosaposin in whole epididymal tissue (A) and luminal fluid (B) of the epididymis from control (Con), castrated (Ct) and castrated with testosterone supplementation (Ct+T) animals. The figure shows a representative immunoblot of each region and experimental condition and the quantification of the bands in each case. Bars represent the means of relative optical density (ROD) ± SE from three independent experiments. No significant differences were noted between (C, Ct and Ct+T). Ponceau staining was used as loading control.

PSAP expression in whole epididymal tissue was evaluated by Western blot immunoblotting in of each of the 3 major regions, i.e. IS/caput, corpus and cauda. Interestingly, no significant difference for PSAP was observed between control and castrated animals with or without testosterone administration (Fig 7A, top panel); levels were comparable to controls as revealed by statistical analysis (Fig 7A, lower panel). Thus, while the LM-IHC quantitative data indicated a significant increase in lysosomal size of principal cells after castration, this did correlate with increased levels of PSAP by western blot analysis.

The expression of PSAP in the epididymal fluid of each of the 3 major regions as analysed by Western blot did not vary in castrated animals with or without testosterone administration (Fig 7B, top panel); levels were comparable to controls as revealed by statistical analysis (Fig 7B, lower panel). Thus, the expression of PSAP in lysosomes of principal cells, as well as its secretion into the lumen, was not modified by absence of testosterone. Moreover, while a qualitatively significant increase in size of lysosomes of principal cells was noted by LM-IHC in both castrated animals with and without testosterone replacement (Fig 2A and 2B), this did not correlate with an increased expression of PSAP in these cells. It is suggested that the size of lysosomes is a result of the adverse effects of castration on the endocytic activity of principal cells, and that this is independent on the expression of PSAP.

### Presence of apoptotic cells in the lumen of castrated animals with and without testosterone replacement

**Fig 8:**
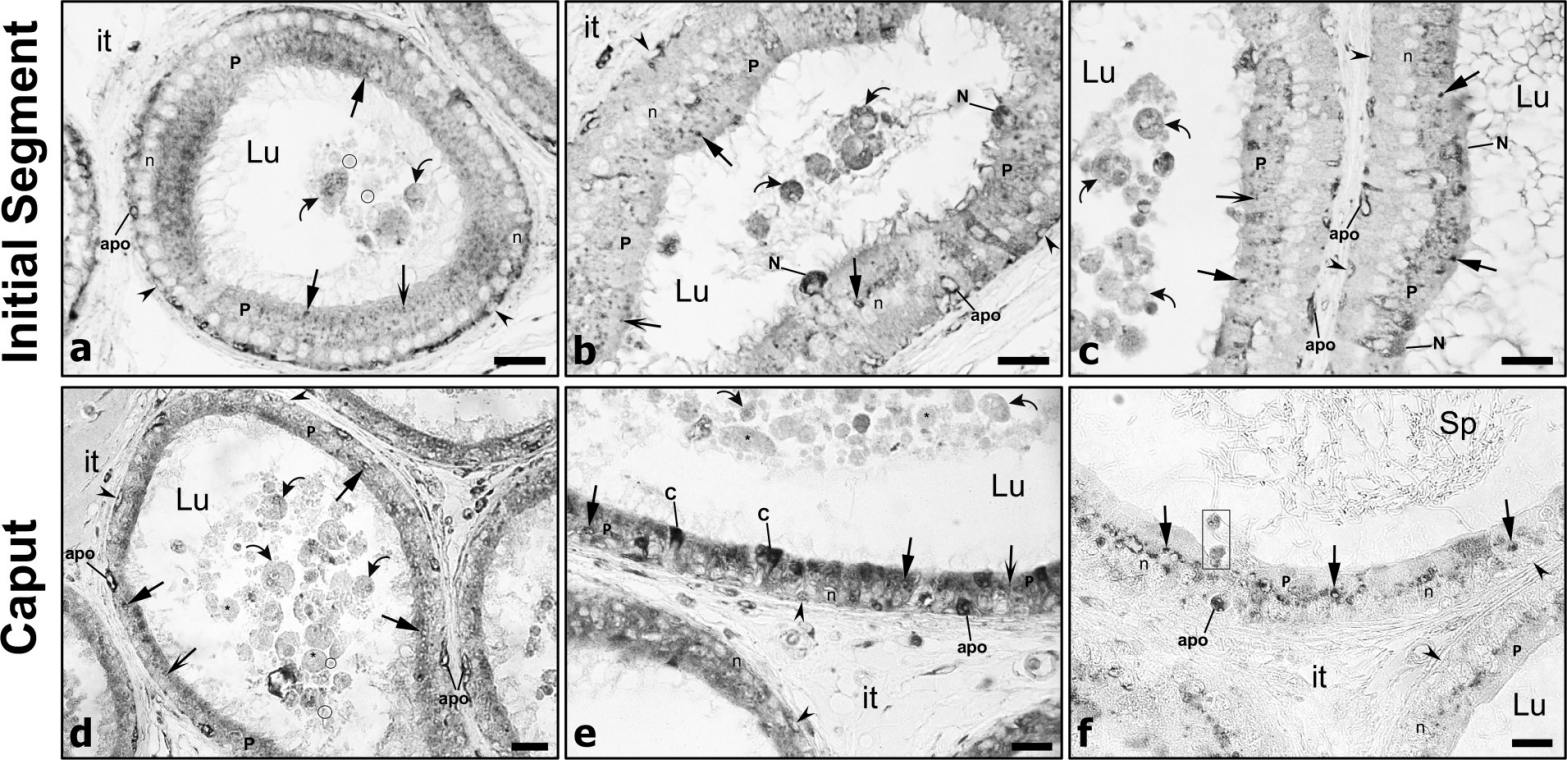
Apoptotic cells in the lumen of castrated animals with and without testosterone replacement. Lumen and epithelium of the initial segment (a-c) and caput epididymidis (d-f) immunostained with PSAP of castrated with testosterone supplementation (a-c) and castrated (Ct) animals (d), and with CatD of Ct animals (e, f). In the lumen a plethora of abnormal structures with diverse shapes, sizes and content are noted, with some revealing a nucleus (curved arrows). Other small and larger structures had a more homogeneous appearance but without much content (asterisks). Many tiny fragments with a homogeneous appearance were also evident in the lumen (circled). Sperm were not evident in the initial segment, but some were seen in the caput region (Sp). In Fig 7F an apopotic cell appears at the base of the epithelium, while other cells appear to be sloughing from the epithelium into the lumen (rectangular box). Overall, the presence of these abnormal structures/cells suggests the apoptotic state of some of the epithelial cells after castration. Lu, lumen; it, interstitial space; reactive basal (arrowheads), narrow (N) and clear (C) cells; n, nuclei of principal cells. Scale bars = 20 μm.

In castrated animals supplemented with (Fig 8A–8C) and without (Fig 8D–8F) testosterone, the lumen of the initial segment (Fig 8A–8C) and caput epididymidis (Fig 8D–8F) revealed a plethora of abnormal structures with diverse shapes, sizes and content. On the whole the luminal structures ranged in size from small to large and were spherical, oblong or irregular in appearance. Several of the spherical structures were enveloped by a plasma membrane and contained a nucleus (Fig 8B–8D). Other small and larger structures had a more homogeneous appearance without apparent content (Fig 8E). Many tiny fragments with a homogeneous appearance were also evident in the lumen (Fig 8A–8E). Sperm were not evident in the initial segment (Fig 8A–8C) but were seen in the caput region (Fig 8F). Apoptotic cells were seen close to the base of the epithelium, while other cellular debris appeared to be sloughing from the epithelium into the lumen (Fig 8F).

### Co-immunoprecipitation of ProCatD with PSAP in the epididymal luminal fluid of castrated rats: Detection of PSAP oligomers

**Fig 9:**
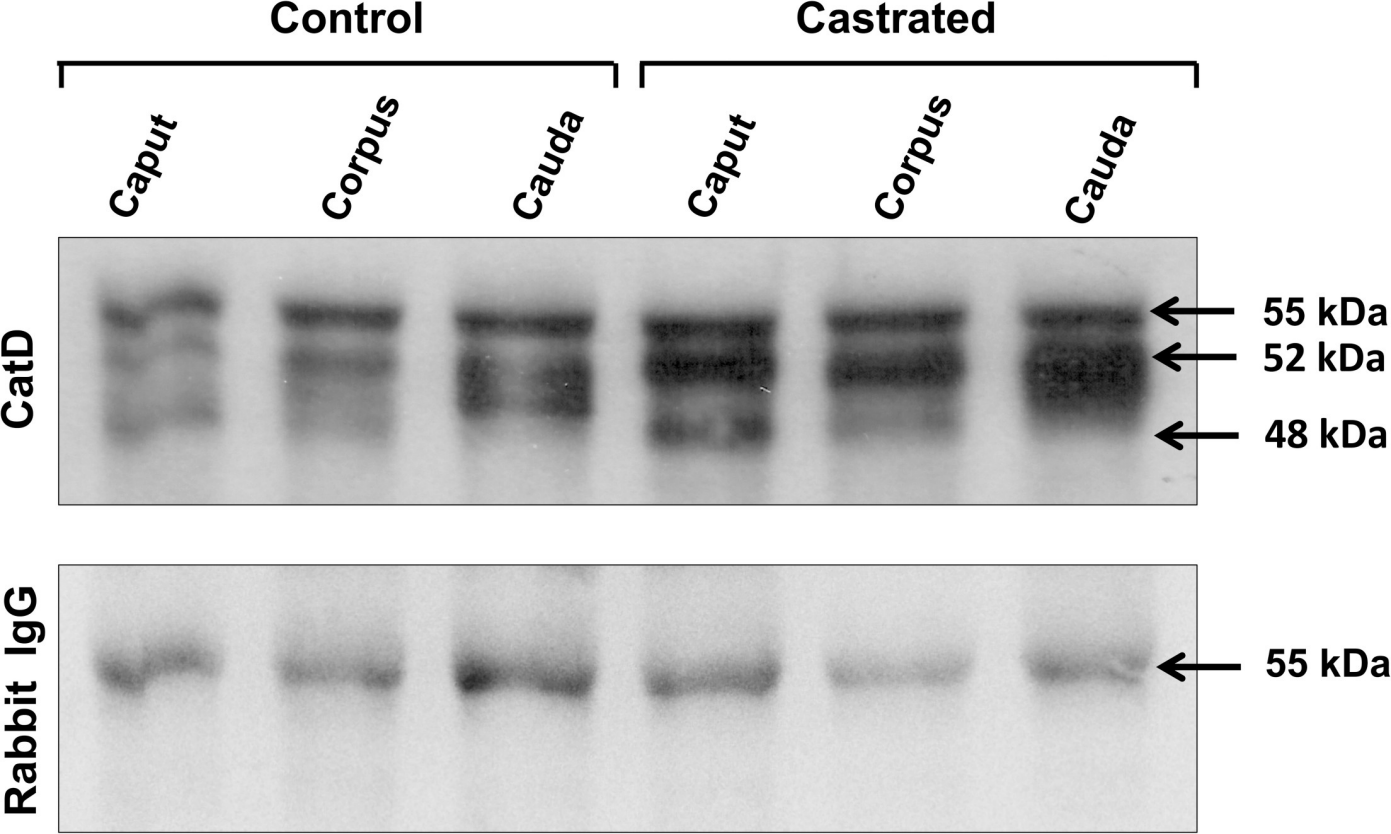
Co-immunoprecipitation of cathepsin D with prosaposin. Epididymal fluid from control versus castrated rats were mixed with beads preincubated with anti-PSAP antibody, as described in Materials and methods. After centrifugation and washing, cathepsin D was detected from the precipitates by immunoblot. Immature (52 kDa) bands of the enzyme were detected in the precipitates. Detection of IgG (55 kDa, heavy chain) was used as a loading control. The figure is representative of two independent experiments.

Further experiments on castrated rats employing co-immunoprecipitation of procatD with PSAP revealed an increase of proCatD secretion into the luminal fluid of the three major epididymal regions (Fig 9). For the first time, it is suggested, that an interaction exists between these proteins in the epididymal fluid and, in turn, the formation of luminal complexes is modified by castration.

**Fig 10:**
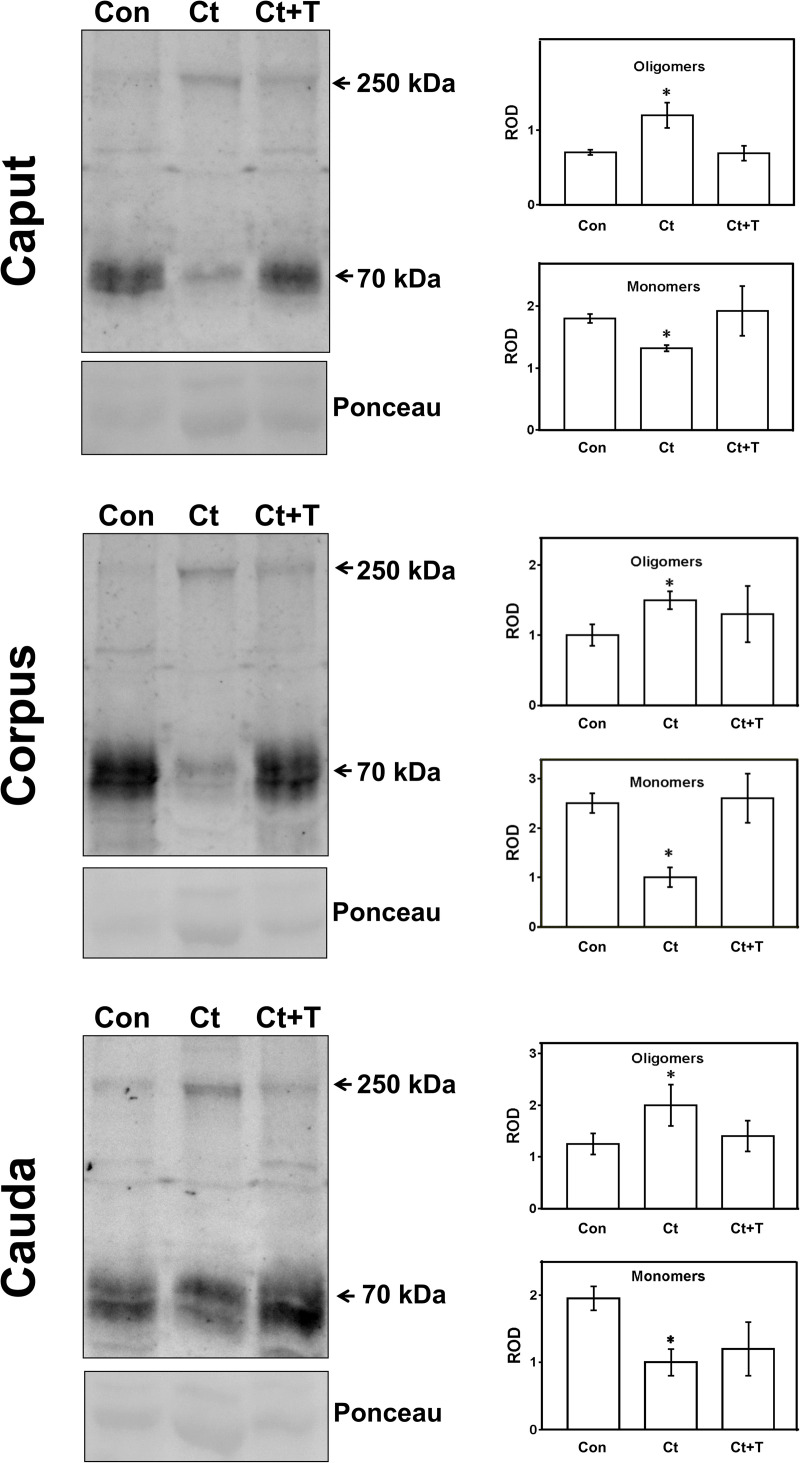
Detection of oligomeric forms of prosaposin in epididymal fluid from control (Con), castrated (Ct) and castrated with testosterone supplementation (Ct+T) animals. Proteins from epididymal fluid were run on SDS-gel under non-reducing conditions and electrotransferred onto nitrocellulose membrane for detection with the specific antibody. Monomers (70 kDa) and oligomers (250 kDa) of PSAP were detected. The figure shows representative immunoblots from each epididymal region and the corresponding quantification of the bands, as indicated. Bars represent the means of relative optical density (ROD) ± SE from three independent experiments. (*) significant differences from the control (p < 0.05, obtained by Tukey’s Multiple Comparisons Test). Ponceau staining was used as loading control.

Based on previous studies on PSAP-proCatD interactions in other tissues [56, 57] and in order to confirm the increased proCatD associated with PSAP after castration, we analyzed by immunoblotting under non-reducing conditions the oligomerization status of PSAP in the epididymal luminal fluid. Notably, we found that a band of ~250 kDa (oligomers) was increased in the three regions of castrated rats consistent with a lower level of monomers (~70 kDa) in these experimental animals (Fig 10, left column). These values were found to be statistically significant in each of the 3 major regions (Fig 10, right column) with the administration of testosterone reversing the situation to normal values. Thus, after castration, an increased oligomerization of PSAP in the epididymal fluid develops in concert with an increased secretion of proCatD into the lumen.

## Discussion

### Quantitative increase in size of lysosomes in principal cells after castration

In the present study, a quantitative analysis confirmed a statistically significant increase in the size of lysosomes of principal cells of castrated animals as compared to controls, as had been suggested over the past decades by many morphological studies [1, 6]. In addition, some of the larger lysosomes showed morphological differences in their appearance exhibiting a dense peripheral rim enveloping a pale central core, which has been documented in EM studies of castrated animals [14].

From many points of view, it has been suggested that the endocytic apparatus develops as a maturation model or as pre-existing compartments employing carrier vesicles between the different endocytic components [58]. In the epithelial cells of the epididymis and efferent ducts, endocytosed products with time appear in early endosomes which then transform in a temporal and sequential manner into late endosomes and finally lysosomes, defining the maturation model of endocytosis [1, 9–11]. Thus, it is reasonable to suggest that endocytosis in principal cells is augmented leading to an enrichment in size of lysosomes due to the accumulation of substrates emanating from the adverse effects of castration. In some respects, these observations are reminiscent of the deleterious effects of lysosomal storage diseases on principal cells [59–61].

Interestingly, the administration of testosterone to castrated animals did not revert the size of lysosomes in principal cells to that of controls, suggesting that the endocytic process is not androgen-regulated.

### Upregulation of CatD in principal cells after castration

Over the years, it has been well established that androgens regulate the expression of many epididymal proteins, which after castration results in their suppression or diminishment [1, 2, 6, 62, 63]. However, in the present study, castration exhibited a statistically significant increase of CatD expression in all 3 regions examined, i.e. the IS/caput, corpus and cauda, by western blot analysis. It is further suggested that CatD enhancement occurs in principal cells, as only these cells revealed dramatic differences in their morphology, and not for that of narrow/apical, clear and basal cells. Several other epididymal-expressed proteins have been demonstrated by different methods to be upregulated after castration [46, 64].

Administration of androgen to castrated animals reversed the situation of increased CatD expression to that of control levels as noted by western blot analysis. Such a finding has also been documented in a multitude of studies on castrated animals examining a diverse population of proteins [1, 6, 47–49, 65]. In the case of the prostate, castration increased the enzyme activity of CatD during the period of rapid prostatic involution, while administration of exogenous testosterone to castrated rats prevented or retarded prostatic weight loss as well as the increase in cathepsin D activity [66]. Interestingly, while androgens reversed the increased CatD expression in all regions after castration to control levels, the increased size of lysosomes in principal cells was not annulled by androgens. These data suggest that the expression of CatD in lysosomes is independent of the endocytic process and that each of these parameters have their own regulatory mechanism which are not necessarily dependent on androgens.

While it is well established that androgens are key regulators of epididymal gene expression [1–3, 6, 46], data from many studies have revealed that neither androgens nor lumicrine factors appear to be sufficient to fully account for the complex coordination of gene expression, and formation of distinct regional microenvironments, along the length of the epididymis [67–70]. Novel candidates that could affect gene expression flexibility are the small non- protein-coding RNAs (sRNAs), a diverse class of molecular regulators [70–73].

Understandably, future experimentation will uncover the various conditions regulating the expression of the wide variety of proteins inhabiting the distinct microenvironments that make up the epididymal duct by analyzing EDL rats.

### Fine tuning of testosterone for CatD expression in principal cells

Our data further suggest that a mechanism exists by which testosterone in control animals regulates the proper levels of CatD expression in principal cells. When enough CatD has been synthesized and targeted to lysosomes in principal cells, at this point testosterone halts its production. In the absence of testosterone, principal cells overproduce CatD, suggesting that the epididymis recognizes that it has been compromised. This finding also implies that the synthesis and targeting of CatD from the Golgi apparatus to lysosomes in principal cells is under the control of testosterone, which defines its proper balance in control animals. In confirmation of this hypothesis, Carvelli et al. [15] observed that expression of cation-dependent MPR (CD-MPR) and cation-independent MPR (CI-MPR) receptors implicated in the targeting of CatD to lysosomes, increased significantly in castrated rats and which were restored to control levels after testosterone administration.

### Castration causes apoptosis of principal and basal cells

One of the underlying themes of the adverse effects of castration is the apoptosis of epithelial cells. The occurrence of this phenomenon as detailed in the literature commences between 6-12h and is completed 3–7 days thereafter, with differences existing between epididymal regions [39–41, 74, 75]. Some investigators suggested that the apoptotic cell death localizes specifically to principal cells [40, 41]. However, more recent studies have demonstrated that a subset of basal cells in addition to principal cells of the initial segment underwent apoptosis 1 day after efferent duct ligation [76, 77]. Thus while our study examines events at 2 days post castration, this time point already marks a defined period when apoptosis is prevalent, and by the fact that the fertility status of sperm is affected as early as 3 days after castration [78].

In the present study, we noted apoptotic cells in the epithelium of the initial segment and caput region at 2 days after castration, in accordance with the peak of such activity in these regions after castration [40, 41]. However, we also noted a plethora of apoptotic debris in the lumen, which could account for the enhanced endocytic activity of principal cells noted in castrated animals with and without androgen replacement and their eventual presence in lysosomes leading to their increased size.

Our LM-IHC analysis also revealed the presence of abnormal degenerating cells at the base of the epithelium and which appeared to represent apoptotic basal cells.

Basal cells have undergone a remarkable resurgence of interest in recent years. While occupying the base of the epithelium of the 4 major regions of the epididymis where they contact the basement membrane, they have been reclassified into distinct cell types [3, 79, 80]. In addition to COX-1/cytokeratin 5 basal cells [3, 81–85], a population of epididymal mononuclear phagocytes (eMPs, monocytes, macrophages and dendritic cells) has been identified at the base of the epithelium [81, 83, 84].

Interestingly, in the prostate, an increase of CatD activity was noted in dendritic cells 4 days after castration [86]. Based on reactivity of basal cells for CatD in the present study, we predict that these reactive cells may include the different basal cell populations which posses phagocytic properties [84], an analysis that would require the use of specific markers and constitute an area for future investigation. In an early EM analysis, Abe and Takano [39] suggested that principal cells underwent degeneration and were digested not only by normal principal cells, but also basal cells. However, the identity of basal cells in their study was not confirmed by specific markers.

### Castration causes increased secretion of proCatD into the epididymal lumen and CatD expression in lysosomes of principal cells in distal epididymal regions

In the mammalian epididymis, proCatD is secreted into the lumen [15, 34, 87] as shown under some physiological and pathological conditions for a variety of different cell types [16, 21, 22]. In the present study, a statistically significant increase in secretion of proCatD of the corpus and cauda regions was observed after castration of samples taken of the epididymal luminal fluid and visualized by western blot and restored after androgen replacement, as was the case for CatD expression in whole epididymal tissue, suggesting that androgens regulate expression and secretion of CatD and proCatD in principal cells of distal regions. While androgens have been proposed to modulate expression of many proteins, we cannot rule out the possibility of an FSH-mediated effect on CatD expression, as the FSH receptor has been identified in the epididymis [88], and since castration produces an increase in FSH, which can be reversed by testosterone replacement [89], an area in need of more work.

While one may consider the role of proCatD and CatD stemming from the apoptosis of epithelial cells after castration in proximal regions, apoptosis in principal cells does not peak until days 5 and 6 in the corpus and cauda regions, respectively [40]. Thus, it may be suggested that specific substrates not arising from apoptotic cells appear in the lumen and lysosomes of principal cells for interaction with these enzymes, which may be derived from abnormal spermatozoa as a consequence of alterations to principal cell functions following castration. The increased CatD expression in the corpus and cauda regions suggests that specific substrates are taken up from the lumen of castrated animals that require CatD processing.

Alternatively, it has been also shown that secreted proCatD can be endocytosed via M6PR or another yet unknown receptor and undergo further maturation, where it would gradually end up in lysosomes for degradation of specific substrates of these enzymes [6, 90].

### Effects of castration on PSAP reactive lysosomes and PSAP secretion

Given that PSAP (sulfated glycoprotein-1) has been implicated in the targeting and activation of CatD [16, 56, 57, 91], the status of PSAP was assessed in the present study. Firstly, castration did not alter the reaction pattern for PSAP of any epithelial cell of the entire epididymis despite the increased lysosomal size. Secondly, western blot analysis of whole epididymal tissue revealed no significant changes in PSAP protein levels between control and castrated animals. This suggests that PSAP expression in principal cells is not regulated by androgens as also demonstrated by Hermo and Andonian [14]. However, from the finding that PSAP is encoded by an androgen-regulated gene [92] the maintenance of PSAP at normal level in epididymis of castrated rats could be due to the small concentrations of androgens secreted by adrenal glands [93]. Nevertheless, the increased CatD expression in principal cells after castration does not appear to be consequential to increased levels of PSAP.

While PSAP localizes to lysosomes of principal cells, it is also present in the luminal fluid of the epididymis, where it assists in modification of membrane lipids during sperm maturation [15, 24]. However, PSAP secretion as noted by immunoblotting is not augmented in castrated animals in any region, unlike the case for proCatD. Indeed many proteins have been also shown to maintain expression after castration and be secreted without androgen stimulation [63, 94]. Interestingly, cathepsins are secreted into extracellular spaces of different tissues following cellular stress, where they have roles in promoting cell survival, differentiation and cleaning up cellular debris following injury [16, 17, 95], which may explain the upregulation of proCatD secretion after castration. The absence of upregulation of PSAP in the lumen and lysosomes of principal cells suggests a lack of enhancement of substrates for this protein after castration.

### Increase in the co-immunoprecipitation of ProCatD with PSAP in the epididymal luminal fluid of castrated rats

The formation of PSAP/ProCatD complexes has long been noted in cell types of different tissues [56, 57, 91]. Indeed it has been shown that fractions of PSAP bound to ProCatD are transported in tandem not only to lysosomes, but also to the extracellular space of certain cell types, and that PSAP forms oligomers that bind proCatD affecting its autoactivation [57]. In the present study, despite the differential response of secreted PSAP and proCatD to hormonal changes, our data revealed an increase in the co-immunoprecipitation of proCatD with PSAP in the epididymal luminal fluid of castrated rats, confirming an affinity between these proteins. The discrepancy could be explained by a higher amount of the proCatD enzyme that is associated with each molecule of PSAP under this experimental condition, since PSAP oligomers have been suggested to have more than one binding site for proCatD [57]. Moreover, castration induced an increased oligomerization of PSAP, by which an interplay between these distinct oligomeric forms could explain the increased affinity for proCatD. Interestingly, oligomerization of PSAP (linked covalently by intermolecular disulphide bridges) is crucial for its entry into the secretory pathway [29].

### Role of saposins and proCatD in the epididymal lumen of control and castrated animals

While it has been documented that PSAP forms oligomers that are capable of binding proCatD spontaneously, and that the expression of the latter plays an important role in the sorting and processing of PSAP to mature saposins [56, 57], then the increased levels of the proCatD could enhance the levels of saposins in the epididymal lumen of castrated rats. Here the saposins could engage in breakdown of their substrates derived from sperm and/or the apoptosis of degenerating epithelial cells, as discussed above.

The role of saposins in the lumen also has relevance under normal conditions. Sulfogalactosylglycerolipid (SGG), major sulfoglycolipid of the sperm plasma membrane has been proposed to be a substrate of the lysosomal enzyme arylsulphatase A (ARSA) [96–98]. ARSA is secreted by the epididymis and binds to the sperm plasma membrane [97, 99]. While the function of ARSA is to modify SGG in relation to the sulfate composition of the sperm plasma membrane, this activity requires the presence of the sphingolipid activator protein saposin B (derived from the lysosomal precursor PSAP) [98]. In the mammalian epididymis, both PSAP and proCatD are secreted into the lumen [15, 24, 87], as observed in the present study. Considering that processing of PSAP to mature saposins requires CatD, then the interaction of these 2 proteins in the lumen could form saposin B, which could then interact with ARSA and modify SGG. Thus, the presence of CatD and PSAP in the epididymal lumen may correlate with the acquisition of sperm maturation and its fertilizing capacity.

In conclusion, our results provide new insights by which hormones regulate the expression of lysosomal proteins that may be related to dysfunctions of the epididymis including male infertility.

## Supporting information

S1 Data Data sheet and statistical analysis used to build graphs in Fig 2. (XLSX)

S2 Data Data sheet and statistical analysis used to build graphs in Fig 4. (XLSX)

S3 Data Data sheet and statistical analysis used to build graphs in Fig 7. (XLSX)

S4 Data Data sheet and statistical analysis used to build graphs in Fig 10. (XLSX)

S1 Raw images Supporting images for Figs 4, 7, 9 and 10. Immunoblottings with respective loading control showing the molecular size marker.
(PDF)
