# Supplementary material for: Castration causes an increase in lysosomal size and upregulation of cathepsin D expression in principal cells along with increased secretion of procathepsin D and prosaposin oligomers in adult rat epididymis
Source: PLoS One. 2021 Apr 29;16(4):e0250454. doi: 10.1371/journal.pone.0250454 (PMC8084160; doi:10.1371/journal.pone.0250454)
Supplement: S1 Raw images — Immunoblottings with respective loading control showing the molecular size marker. (PDF) [file pone.0250454.s005.pdf]

Supporting images for Figures 4, 7, 9 and 10

Abbreviations:

MWM: molecular weight marker
Con: control
Ct: Castrated
Ct+T: Castrated with testosterone supplementation
X: lane not included in the final figure

Figure 4

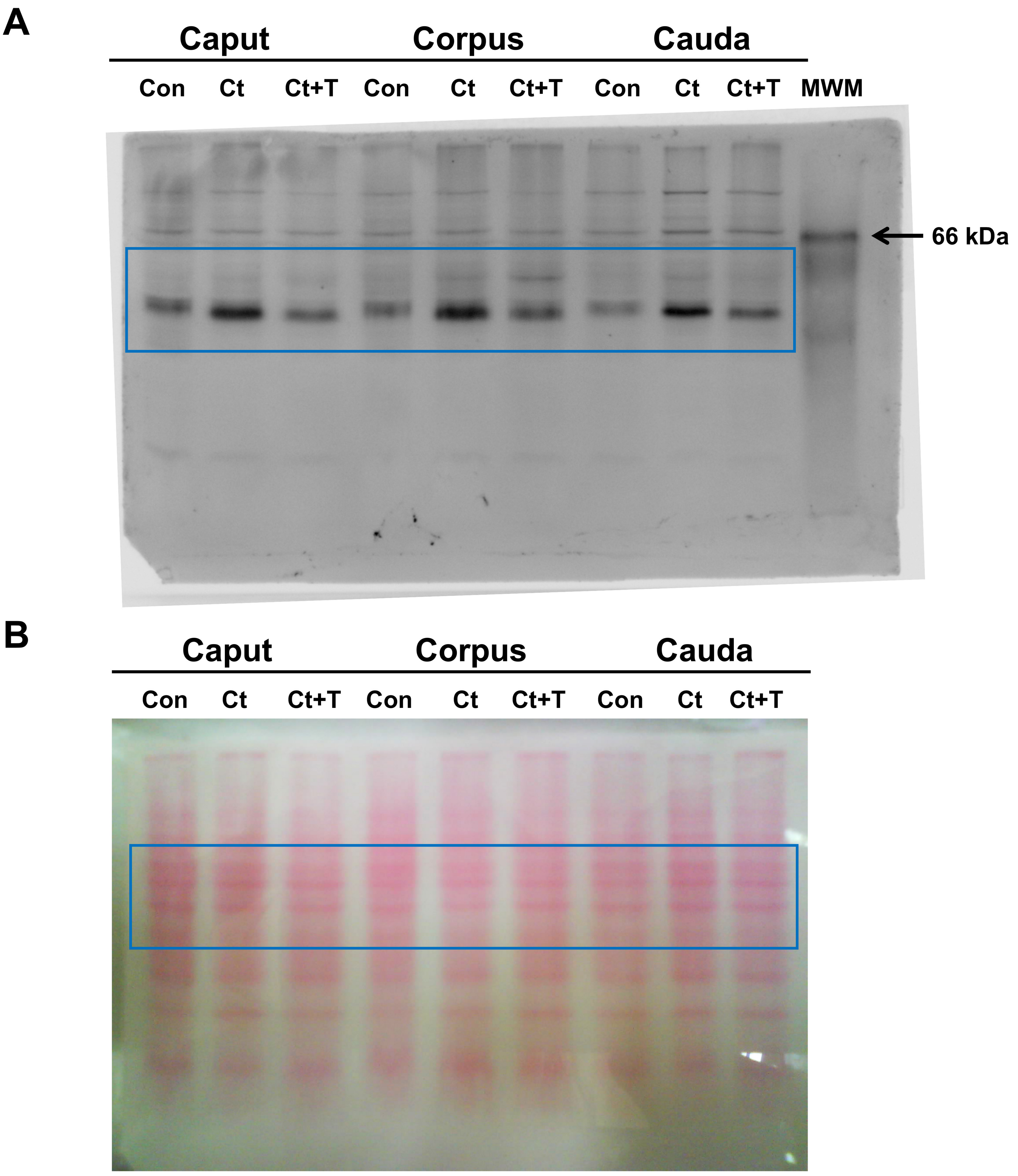

Immunoblotting of cathepsin D (**A**) showing the molecular size marker (biotinylated bovine serum albumin) and its respective loading control (**B**). Image **A** was captured with LAS 4000 imaging system (Fujifilm Lifescience, USA). Image **B** was captured with a Canon Powershot SD1100IS camera.

Figure 7

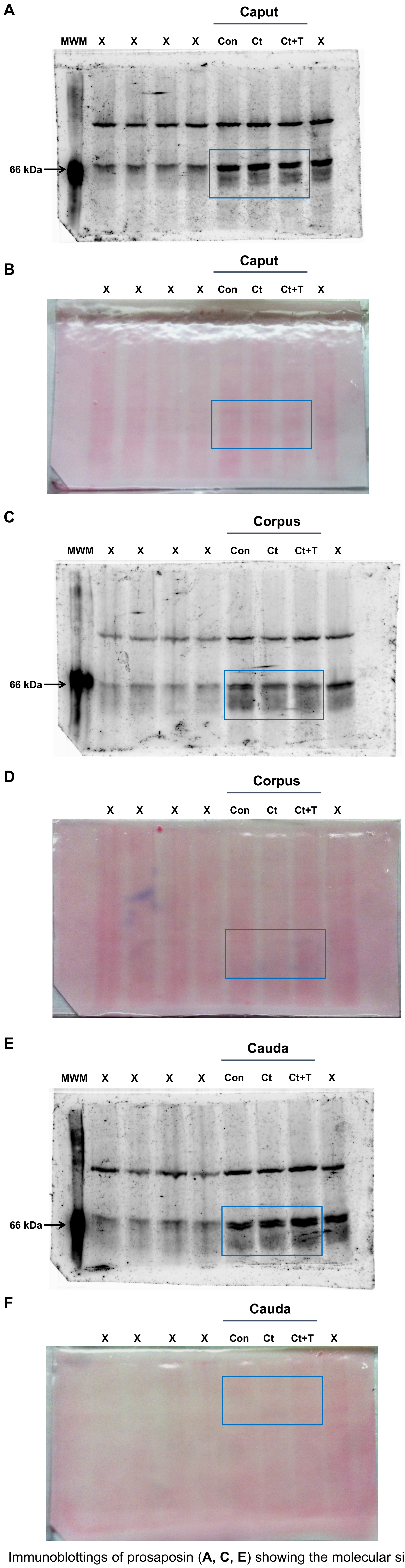

Immunoblottings of prosaposin (**A, C, E**) showing the molecular size marker (biotinylated bovine serum albumin) and their respective loading controls (**B, D, F**). Images **A, C** and **E** were captured with LAS 4000 imaging system (Fujifilm Lifescience, USA). Images **B, D** and **F** were captured with a Canon Powershot SD1100IS camera.

Figure 9

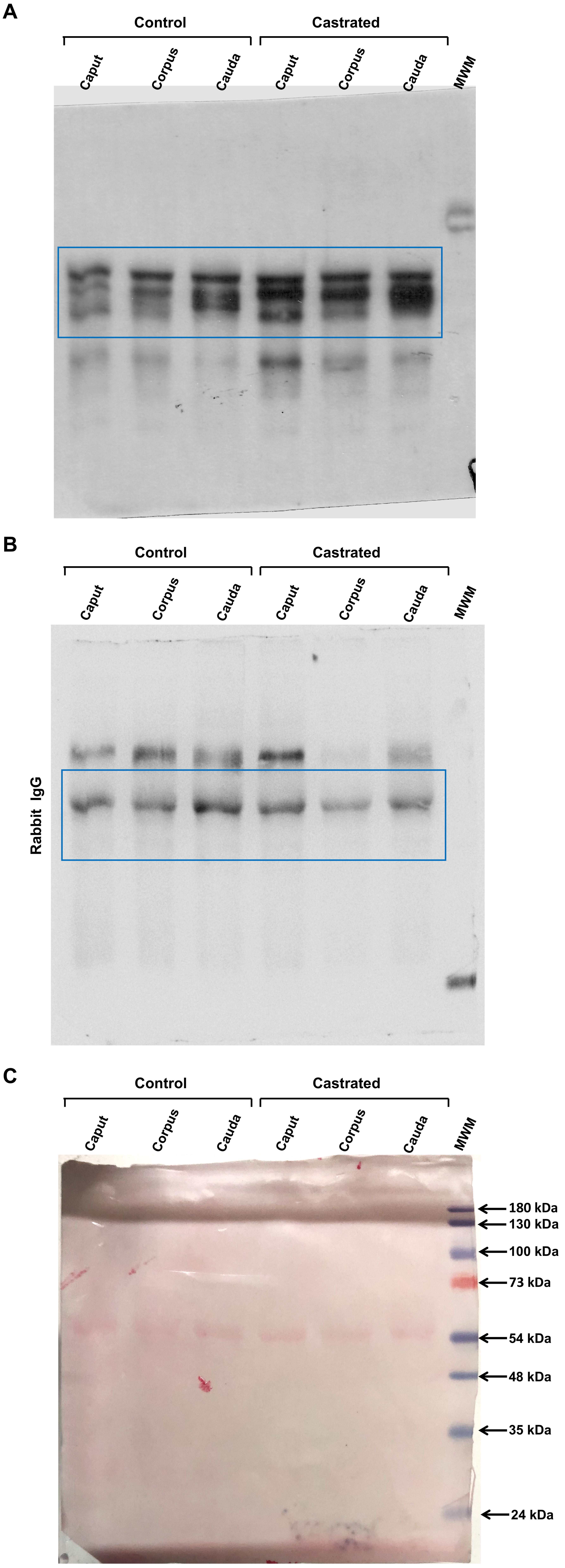

Immunoblotting of cathepsin D (**A**) with its respective loading controls (**B** and **C**) showing the molecular size marker. Images **A** and **B** were captured with LAS 4000 imaging system (Fujifilm Lifescience, USA). Image **C** was captured with a Canon Powershot SD1100IS camera.

Figure 10

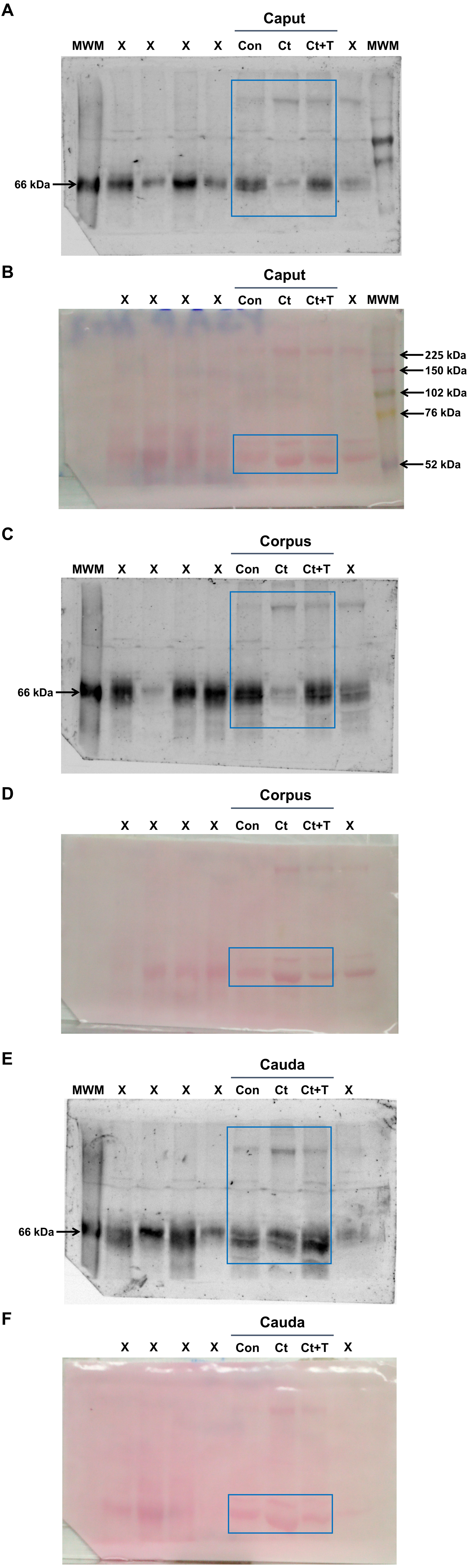

Immunoblottings of prosaposin (**A, C, E**) showing the molecular size marker (Amersham™ ECL™ Rainbow™ marker and/or biotinylated bovine serum albumin) and their respective loading controls (**B, D, F**). Images **A, C** and **E** were captured with LAS 4000 imaging system (Fujifilm Lifescience, USA). Images **B, D** and **F** were captured with a Canon Powershot SD1100IS camera.
